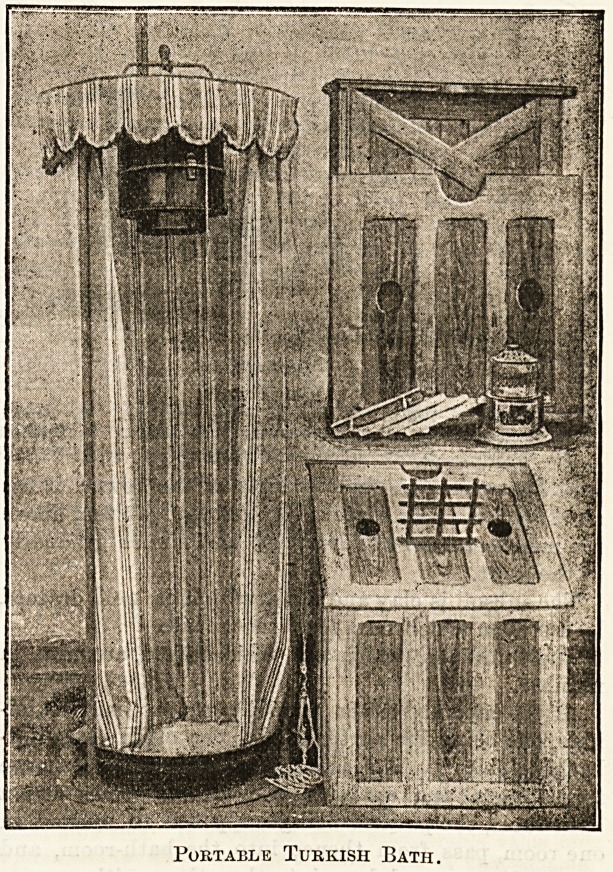# Practical Departments

**Published:** 1894-12-01

**Authors:** 


### PRACTICAL DEPARTMENTS.

### COMBINATION TURKISH BATH.

At the Laundry and Sanitary Exhibition recently held at the Agricultural Hall might have been noticed, somewhat hidden from view by larger exhibits on either hand, an arangement which purports to be nothing less than a Portable Turkish Bath, complete in every particular, and a closer examination shows that this combination bath really fulfils all the conditions required to make it a very efficient and convenient substitute for the more elaborate process usually associated with the name.

The illustration which we give, by kind permission of the inventor, Mr. Walford, of Dallinghoo, Suffolk, shows pretty clearly of what the apparatus consists. The hot-air chamber is composed of four folding sides, with a lid, and is made of unvarnished wood. The bather enters through a small door in front, which is then fastened. The lid, which is in one piece, can be easily drawn up and secured from inside, and, it is seen, has holes for the head and arms, those for the latter being closed when wished by sliding shutters inside. A book-rest is supplied so that the bather can read with comfort. Instead of a hard seat a comfortable hammock seat is slung across the chamber, and is adjustable to any height required.

The heating is accomplished by means of a methylated spirit lamp, to which is fitted a vessel for holding water should a vapour bath be desired. The lamp is movable, so that it may be placed wherever the bather pleases. A special point to be noted is that the hot-air chamber is raised from the ground about half an inch on castors, so that a constant influx of fresh air is secured, feeding the lamp and the body, the lungs breathing in pure air all the while the bather is in the hot-air chamber. There is plenty of room inside. We have said that the chamber has folding sides. The doors and sides fold inwards, the whole apparatus, when folded and placed against the wall, having the appearance of a small cupboard, and only occupying a floor space of some 3 feet by 6 inches.

But this is only a portion of the process. Most substitutes for the Turkish bath have hitherto failed, because of the difficulty of securing in private houses shower baths of graduating temperature. This obstacle has been overcome in the portable shower-bath, which is a part of Mr. Walford's apparatus, and which is so contrived as to supply showers of three variations in temperature. The cistern is a double one, and is lowered to be filled by means of pulleys. Hot water is poured into the top receptacle, and through a valve descends into the lower one. The upper one is then filled with cold water, and the cistern returned to its position. By an ingenious arrangement of chains and levers the bather, on entering the shower bath after getting out of the hot chamber, pulls down upon himself first a plentiful shower of hot water, then by touching another chain the remaining hot water in the upper division mingles with the cold in the lower, and descends in a tepid stream, and finally on pulling both chains

Portable Turkish Bath.

together a cold shower completes the process. It should be mentioned that the sponge bath attached is not in any way fixed, and can therefore be used independently.

Mr. Walford's apparatus is one which should be found of much value by those who habitually and systematically take Turkish baths. Many people find the greatest benefit by so doing, and in such cases no plan could be more convenient or satisfactory than to have the means at hand with little or no trouble. An apparatus which can be put so easily away when not in use has also much to recommend it. For use in hospitals as well as private houses, it will no doubt be found really valuable.

The price of the whole apparatus complete is twelve guineas, and it is manufactured by Messrs. A. Blake and Sons, Dallinghoo, Wickham Market, Suffolk, from whom it may be obtained.

## Figures and Tables

**Figure f1:**